# Hyaluronic Acid (800 kDa) Supplementation of University of Wisconsin Solution Improves Viability of Osteochondral Grafts and Reduces Matrix Metalloproteinase Expression during Cold Preservation

**DOI:** 10.1155/2015/631369

**Published:** 2015-06-25

**Authors:** Takuya Yamada, Kentaro Uchida, Kenji Onuma, Gen Inoue, Jun Aikawa, Shotaro Takano, Hiroyuki Sekiguchi, Hisako Fujimaki, Masayuki Miyagi, Masashi Takaso

**Affiliations:** ^1^Department of Medical Engineering and Technology, School of Allied Health Science, Kitasato University, 1-15-1 Minami-ku, Kitasato, Sagamihara, Kanagawa 252-0374, Japan; ^2^Department of Orthopedic Surgery, Kitasato University School of Medicine, 1-15-1 Minami-ku, Kitasato, Sagamihara, Kanagawa 252-0374, Japan

## Abstract

Osteochondral allografting is a promising option for the treatment of large cartilage defects. However, because the cell viability of osteochondral tissues (OCTs) gradually reduces during storage at 4°C, methods for maintaining the cell viability of fresh OCTs are needed to improve transplantation outcomes. Here, we evaluated whether the supplementation of preservation solution with one of three different molecular weight forms of hyaluronic acid (HA) improved the viability of rat OCTs during long-term cold storage. The supplementation of University of Wisconsin (UW) solution with 800 kDa significantly improved the cell viability of OCT after 14 days at 4°C compared to nonsupplemented UW solution. In contrast, UW solution supplemented with either 1900 or 6000 kDa HA did not markedly improve the cell viability of the OCT. Real-time PCR analysis revealed that the levels of matrix metalloproteinases 2, 3, and 9 were significantly decreased in OCT stored in UW solution supplemented with 800 kDa HA. Although further studies in human OCT are warranted, these findings demonstrate that the use of 800 kDa HA in place of serum may be a suitable approach for the long-term preservation of osteochondral allografts designated for the repair of large cartilage defects in the clinical setting.

## 1. Introduction

Osteochondral allografting is promising option for the restorative treatment of large cartilage defects caused by osteonecrosis, osteochondritis dissecans, and traumatic injury due to the low rates of donor-site morbidity. Although fresh osteochondral allografts typically have high functional outcomes after transplantation [1], the clinical use of this tissue is associated with the possibility of disease transmission. For this reason, OCTs are typically preserved at 4°C during the required 14-day disease-screening period. However, the cell viability of OCTs gradually decreases during cold preservation, leading to marked reductions in the efficacy and clinical outcomes of cartilage repair [2]. Therefore, maintaining a high cell viability of OCT during cold storage in culture medium is necessary to improve the efficacy of osteochondral allografting.

The viability of chondrocytes in OCTs is markedly enhanced by the supplementation of storage medium with fetal bovine serum (FBS) [3, 4]. However, due to the potential risk of human infection associated with the use of FBS, the development of serum-free methods that allow for the long-term storage of allogenic grafts is desirable. One potential approach is the use of University of Wisconsin (UW) solution, which as we previously demonstrated was superior to culture medium (Dulbecco's Modified Eagle Medium (DMEM)) for maintaining OCT viability [5]. However, because OCTs stored in serum-free UW solution have relatively low cell viability [6], it is necessary to identify a non-animal-derived component that promotes the cell viability of OCT during long-term storage.

Hyaluronic acid (HA), which is a major component of the extracellular matrix of cartilage, is widely used to treat osteoarthritis by intra-articular injection. Animal and clinical studies have shown that HA has antiapoptotic and anti-inflammatory effects on chondrocytes and can slow the degenerative processes of osteoarthritis [7–10]. These properties suggest that HA has the potential to improve the cell viability of OCT during cold preservation.

Here, we investigated whether the supplementation of UW solution with HA increases the cell viability of rat OCT during long-term cold storage.

## 2. Materials and Methods

### 2.1. Preparation of UW Supplemented with HA

Three different molecular weight forms of HA used clinically for the treatment of OA in Japan were used in study: 800 kDa HA (ARTZ), which was purchased from Kaken Pharmaceutical Co., Ltd. (Tokyo, Japan); 1900 kDa HA (Suvenyl), which was purchased from Chugai Pharmaceutical Co., Ltd. (Tokyo, Japan); and 6000 kDa HA (Synvisc), which was purchased from Teijin Pharma, Ltd. (Tokyo, Japan). The concentration of HA used in the UW solution was 0.1% and was selected based on the preliminary findings of a pilot study.

### 2.2. Preparation of OCT Samples

All animal experiments were performed in accordance with the guidelines of the Animal Ethics Committee of Kitasato University. To collect OCT, 52 male 14-week-old Sprague-Dawley rats (Charles River Japan, Inc., Yokohama, Japan) were initially anesthetized with diethyl ether and were then fully anesthetized by the intramuscular injection of a mixture of medetomidine, midazolam, and butorphanol tartrate. A skin incision was made just above the knee, and the distal femoral condyles were exposed by cutting the patellar, cruciate, and collateral ligaments. After removing the distal parts of the knee extensor muscles from the bone, a bone saw was used to cut the distal femur in the metaphyseal region. Two OCT samples were harvested from the bilateral knees of each rat, and the collected OCT samples were placed into separate tubes and then adjusted to approximately 300 mg wet weight using a scalpel or rongeur to remove soft tissue and bone tissue. Pooled OCTs from the left (*n* = 20) and right knees (*n* = 20) were each divided randomly into two groups of 10 OCTs to avoid two OCTs from one animal being included in the same group. UW solution alone (UW), UW solution supplemented with 800 kDa HA (HA800), UW solution supplemented with 1900 kDa HA (HA1900), and UW solution supplemented with 6000 kDa HA (HA6000) were evaluated for the measurement of cell viability and histological assessment. The cell viability of 10 OCT samples without cold preservation from the right knee of rats was also used as controls for the measurement of cell viability. In addition, the OCT samples obtained from the left and right knees of 6 rats were separated into 2 groups (*n* = 6/group), UW and HA800, for real-time PCR analysis.

### 2.3. Measurement of Cell Viability in Preserved OCTs

Cell viability analysis for OCT was performed using the water-soluble tetrazolium (WST) assay with a commercial WST kit (Cell Count Reagent SF; Nacalai Tesque, Kyoto, Japan), as described previously [5, 6], after 14 days of preservation at 4°C, because rat OCT has markedly reduced viability after this time point [6]. For the assay, nonpreserved (baseline group) and cold-preserved OCT samples in UW solution with or without HA supplementation were incubated in 3 mL DMEM containing 10% WST assay reagent at 37°C for 2 h. The culture supernatant was then transferred to 96-well plates for the measurement of absorbance at 450 nm using a SpectraFluor Plus multiple plate reader (Tecan, Männedorf, Switzerland). For each treatment group, the absorbance values of 10 OCT samples were averaged, and cell viability was then calculated from the dye absorbance versus OCT sample quantity. In this study, cell viability is expressed relative to the absorbance of the nonpreserved OCT samples (100%).

### 2.4. Histological Assessment

After assaying the cell viability of OCTs, morphological features of cells in the articular cartilage of OCT samples were assessed as previously described [6, 11]. Briefly, OCT samples were fixed in 4% paraformaldehyde phosphate buffer for 48 h at 4°C and were then decalcified in 0.5 M EDTA for 2 weeks at 4°C. The samples were embedded in paraffin, and 3 *μ*m thick sagittal sections of the patellar groove of femoral condyles were prepared and stained with hematoxylin and eosin. The thickest site of articular cartilage in noncalcified zones was readily observed at 200x magnification using a light microscope. Each set of ten tissue specimens prepared from OCT samples preserved in UW solution supplemented with or without HA was analyzed using a computer-assisted imaging system (Flovel, Tokyo, Japan). Pearsall IV et al. [12] histologically examined cold-preserved human osteochondral allografts and found that the severe pyknosis of chondrocytes and characteristic changes in cell shape were indicative of nonviable chondrocytes. In addition, we previously reported that pyknotic nuclei and shrunken eosinophilic cytoplasm are a characteristic feature of degenerative chondrocytes [6]. Therefore, to determine the proportion of degenerative chondrocytes in each sample, the number of chondrocytes with normal morphological features (normal chondrocytes) and pyknotic and irregular nuclei or severe pyknotic and shrunken eosinophilic shrunken cytoplasm (degenerative chondrocytes) was determined and divided by the total number of chondrocytes in noncalcified zones. Safranin-O staining was also performed for the assessment of glycosaminoglycan. The stained samples were evaluated according to Mankin's histological-histochemical grading system (normal, slight reduction, moderate reduction, severe reduction, and no dye noted), as previously described [6].

### 2.5. Real-Time PCR

Total RNA was extracted from OCT samples using TRIzol reagent (Invitrogen, Carlsbad, CA), as directed by the manufacturer, and was then used as template for first-strand cDNA synthesis with SuperScript III RT (Invitrogen). Quantitative PCR was then performed using a Real-Time PCR Detection System (CFX-96; Bio-Rad, CA, USA) and 25 *μ*L reaction mixtures consisting of 2 *μ*L cDNA, specific primer set (0.2 *μ*M final concentration; Table 1), and 12.5 *μ*L SYBR* Premix Ex Taq* (Takara, Kyoto, Japan). The PCR cycle parameters were as follows: denaturation at 95°C for 1 min, followed by 40 cycles of 95°C for 5 sec and 60°C for 30 sec. mRNA expression was normalized to the levels of GAPDH mRNA.

## 3. Results

### 3.1. Histomorphometric Analysis

**Figure 1:**
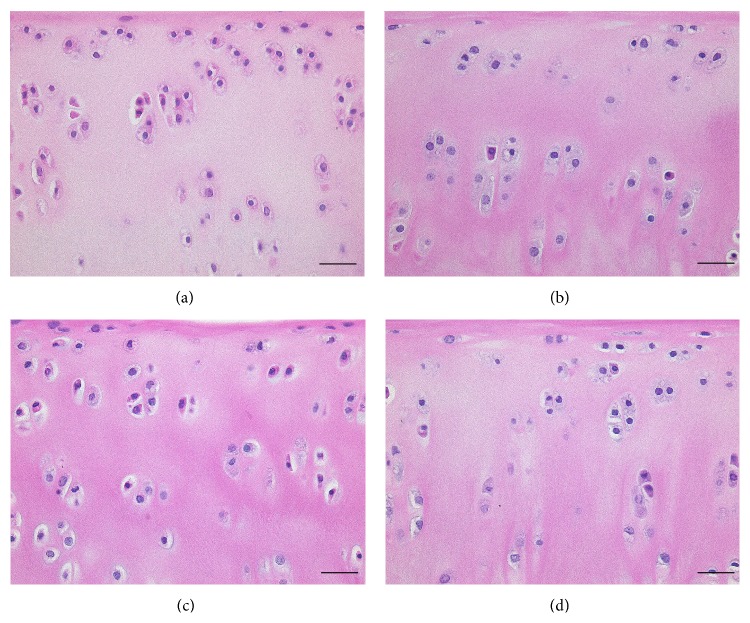
Representative hematoxylin and eosin stained tissue sections of OCTs cold preserved in the presence and absence of hyaluronic acids. Histological analysis of OCTs was performed after 2 weeks of preservation in UW (a), UW with HA800 (b), UW with HA1900 (c), and UW with HA6000 (d). Scale bar, 50 *μ*m.

**Figure 2:**
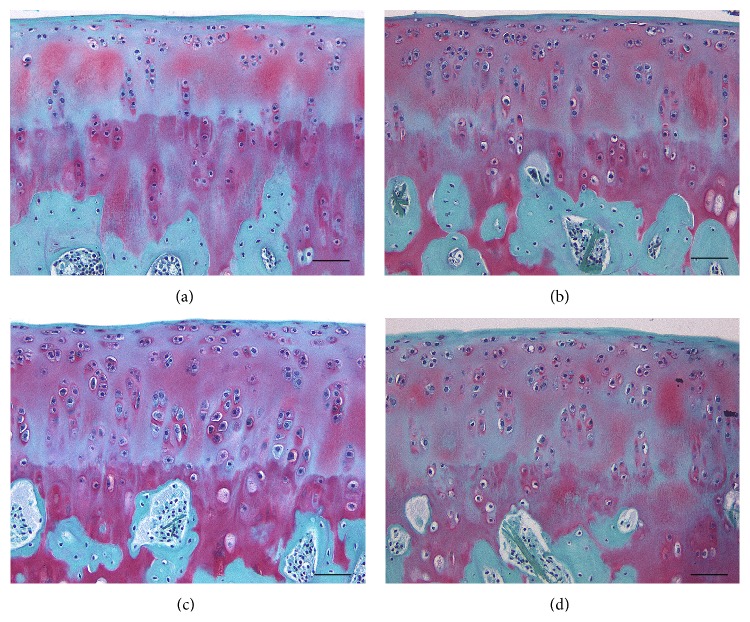
Representative Safranin-O stained tissue sections of OCTs after cold preservation in the presence and absence of hyaluronic acid. Safranin-O staining of OCTs was performed after 2 weeks of preservation in UW (a), UW with HA800 (b), UW with HA1900 (c), and UW with HA6000 (d). Scale bar, 100 *μ*m.

To determine the proportion of degenerative chondrocytes in OCTs after long-term cold storage, sections of OCTs prepared after 14 days of cold preservation in UW solution supplemented with and without HAs were hematoxylin and eosin stained and analyzed by light microscopy (Figure 1). After 14 days of storage in UW solution supplemented with HA800 and HA6000 at 4°C, the OCT samples had significantly more normal chondrocytes and fewer degenerated chondrocytes compared with those stored in UW solution without HA800 or HA6000 (*P* < 0.05; Table 2). The mean proportions of normal chondrocytes in OCTs preserved in UW solution alone and UW solution supplemented with HA1900 were 3.3% and 5.9%, respectively, whereas those in UW solution supplemented with HA800 and HA6000 were 22.4% and 15.8%, respectively. The mean proportions of degenerated chondrocytes in OCTs preserved in UW solution alone and UW solution supplemented with HA1900 were 96.7% and 94.2%, respectively, whereas those in UW solution supplemented with HA800 and HA6000 were only 77.6% and 84.2%, respectively. In addition, Safranin-O staining revealed that only a slight reduction in glycosaminoglycan levels occurred in OCTs preserved UW solution supplemented with HA800, HA1900, and HA6000 compared to the moderate reduction in glycosaminoglycan levels that was observed in OCTs stored in UW solution alone (Figure 2).

### 3.2. Effect of HA on OCT Viability after Cold Preservation

**Figure 3:**
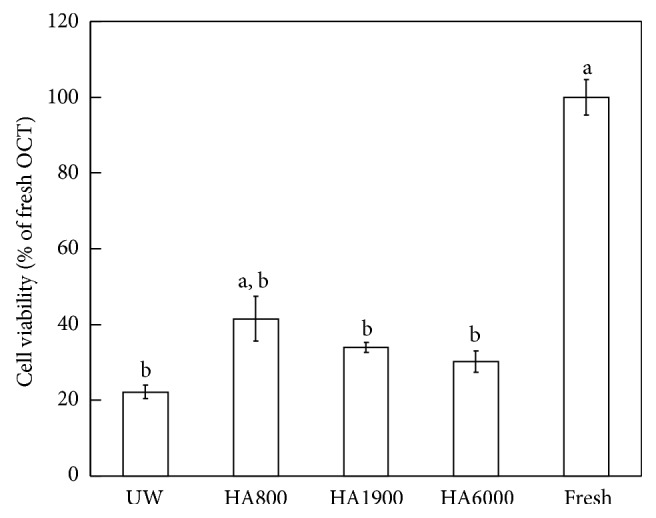
Effect of hyaluronic acid on cell viability in OCT after cold preservation for two weeks in UW solution. Data are presented as the mean ± SE (*n* = 10). ^a^Significant difference between the UW and UW with HA groups (*P* < 0.05). ^b^Significant difference between the nonpreserved OCT and UW and UW with HA groups (*P* < 0.05). Fresh: nonpreserved OCT samples.

As determined using the WST assay, the supplementation of UW solution with HA800 significantly improved the cell viability of OCTs after 14 days at 4°C compared to nonsupplemented medium (Figure 3). In contrast, UW solution supplemented with either HA1900 or HA6000 did not markedly improve the cell viability of the OCT. These findings indicated that UW solution supplemented with HA800 increased the cell viability of OCTs and reduced the degeneration of chondrocytes during long-term cold storage.

### 3.3. Real-Time PCR Analysis

**Figure 4:**
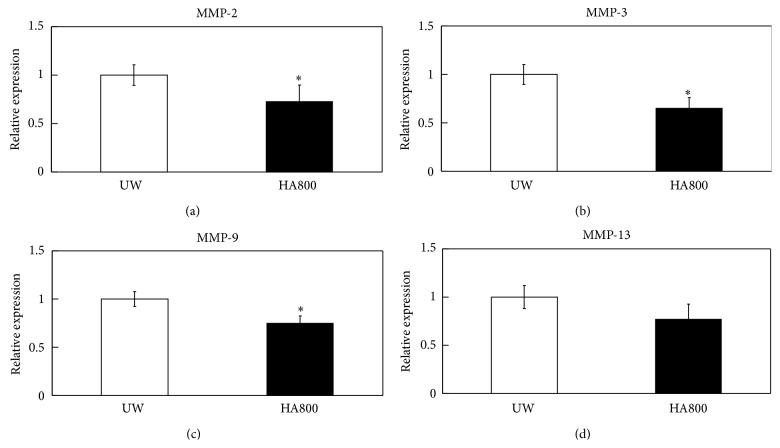
Real-time PCR analysis of OCTs cold preserved in the presence and absence of hyaluronic acid. Expression of MMP-2 (a), MMP-3 (b), MMP-9 (c), and MMP-13 mRNAs (d) in OCT stored for two weeks in UW solution (UW) and UW solution supplemented with 800 kDa HA (HA800). ^*∗*^Indicating a statistically significant difference between the UW and UW with HA800 groups. All data are shown as the mean ± SE (*n* = 6).

To investigate the mechanism by which HA800 increased the cell viability of OCT after cold preservation, OCTs stored in UW supplemented with and without HA800 for 14 days were analyzed by real-time PCR for the expression of several matrix metalloproteinase (MMP) genes (Figure 4). MMP genes were selected because their expression levels are increased in degenerative chondrocytes [13–15] and in livers in response to cold stress [16]. The real-time PCR analysis showed that expression of the genes encoding MMP-2, -3, and -9 was significantly decreased in OCTs stored in UW supplemented with HA800 compared to those in UW alone (Figures 4(a)–4(c)). Expression of the MMP-13 gene was also markedly decreased in OCTs stored in HA800-supplemented UW solution compared to those stored in UW alone, although the difference was not statistically significant (Figure 4(d)).

### 3.4. Statistical Analysis

Differences among the UW, HA800, HA1900, and HA6000 groups were examined using one-way ANOVA with Tukey's multiple comparison test. Student's *t*-test was used to examine differences in gene expression between the UW and HA800 groups. All statistical analyses were performed using SPSS software (Version 11.0; SPSS, Inc., Chicago, IL). A *P* value of <0.05 was considered statistically significant.

## 4. Discussion

In this study, the supplementation of UW solution with HA800 significantly increased the cell viability of OCTs during long-term cold preservation at 4°C. Notably, far fewer degenerated chondrocytes were detected in stored OCTs in comparison with OCT stored in UW solution alone and UW solution supplemented with higher molecular weight forms of HA. These findings show that UW solution supplemented with HA800 kDa is a promising medium for the storage of osteochondral allografts designated for the repair of large cartilage defects in the clinical setting.

HA modulates a number of biological processes, including cell apoptosis and inflammation. It has been demonstrated that these biological effects differ depending on the molecular weight of HA [17], although the underlying mechanisms are poorly understood. In the present study, three different molecular weight forms of HA that are clinically approved were evaluated for their potential to improve OCT quality during cold storage in UW solution. The supplementation of UW solution with HA800 improved the cell viability of OCTs approximately 2-fold compared to unsupplemented UW solution. As HA800 is approved for the treatment of osteoarthritis and because UW solution is approved for organ preservation, UW supplemented with HA800 is a promising solution for the cold preservation of OCTs in the clinical setting.

In degenerative joints, cartilage matrix is degraded by the action of proteinases. Proteins of the MMP family are mainly responsible for cartilage degradation and are often increased in osteoarthritic cartilage [13–15]. However, it has been demonstrated that HA reduces the reduction and activity of MMPs [18, 19]. For example, Julovi et al. [18] reported that HA800 inhibits the activities of IL-1*β*-induced MMP-3 and MMP-13 in cartilage. Consistent with that finding, here, we found that the supplementation of UW solution with HA800 significantly reduces MMP-2, -3, and -9 expression in OCTs during cold storage compared to UW alone. Previous studies also demonstrated that the levels of MMP-2 and MMP-9 are elevated in degenerative chondrocytes [13, 15] and are also increased in the liver and in response to cold stress during preservation [16]. In addition, MMP activity is greater when liver tissue is stored in a relatively poor preservation solution, such as Eurocollins solution, compared to the activity levels in UW solution [16]. Notably, supplementation of the organ preservation solution with MMP inhibitor or knockout of MMP-9 in mice improved the cell viability of the organ tissue during cold storage [20–22]. The present real-time PCR analyses also demonstrated that HA800 reduces MMP-2 and MMP-9 expression in OCTs after 14 days of storage at 4°C. Although further studies are needed to determine the underlying mechanisms, the present findings suggest that HA800 improves OCT viability through the inhibition of MMP production.

In conclusion, the supplementation of UW solution with HA800 is a promising approach for maintaining the cell viability of OCT. Although the effects of HA800 on chondrocyte viability need to be confirmed in human OCT, the use of HA800 in place of serum may be a suitable approach for the long-term preservation of osteochondral allografts used for cartilage repair in the clinical setting.

## Figures and Tables

**Table 1:** Sequences of the primers used in this study.

Gene	Direction	Primer sequence (5′-3′)	Product size (bp)
MMP-2	F	GTTTCCGCTGCATCCAGACT	149
	R	GGGCTCAGGGTCTCATAACG	

MMP-3	F	TTGGCACAAAGGTGGATGCT	103
	R	TGGGTCACTTTCCCTGCATT	

MMP-9	F	CGTGACCTATGACCTCCTGC	229
	R	TAAAGGTTGGGGGATCCGTG	

MMP-13	F	AGGCCTTCAGAAAAGCCTTC	298
	R	TCCTTGGAGTGATCCAGACC	

GAPDH	F	TGC CAC TCA GAA GAC TGT GG	129
	R	TTC AGC TCT GGG ATG ACC TT	

**Table 2:** Proportion of normal and degenerative chondrocytes in OCT samples cold preserved in hyaluronic acid- (HA-) supplemented UW solution.

Storage solution	Normal (%)	Degenerative (%)
UW	3.3 ± 1.0	96.7 ± 1.0
UW + HA800	22.4 ± 4.5^*∗*^	77.6 ± 4.5^*∗*^
UW + HA1900	5.8 ± 1.1	94.2 ± 1.1
UW + HA6000	15.8 ± 2.9^*∗*^	84.2 ± 2.9^*∗*^

^*∗*^ *P* < 0.05.
